# An Ecological Model of Risk Factors in Older Adults With Repeated Exposure to Elder Mistreatment

**DOI:** 10.1093/geroni/igab046.1652

**Published:** 2021-12-17

**Authors:** Mengting Li, Qun Le, XinQi Dong

**Affiliations:** 1 Rutgers, The State University of New Jersey, New Brunswick, New Jersey, United States; 2 Rutgers University, New Brunswick, New Jersey, United States; 3 Rutgers University, Rutgers Institute for Health, New Jersey, United States

## Abstract

Limited empirical studies examined the factors related to repeated EM exposures among Chinese older immigrants. Guided by the ecological model, this study aims to explore what are the risk factors leading to recurrence of EM. Data were drawn from the two-wave PINE Study with 725 participants having EM at baseline and 191 reported repeated EM after two years. EM was evaluated by a 66-item instrument, including psychological, physical, and sexual mistreatment, financial exploitation, and caregiver neglect. Logistic regression was used. Increasing financial independence was associated with lower possibility of repeated EM (OR: 0.72, 95%CI 0.56-0.92). Every one unit increase in ADL impairment (OR: 1.10, 95%CI 1.02-1.18), IADL impairment (OR: 1.09, 95%CI 1.05-1.13) and increase frequency of alcohol consumption (OR: 1.33, 95%CI 1.06-1.66) were associated with higher possibility of repeated EM. Social service could improve physical function, provide financial support, and reduce health-risk behavior to prevent the recurrence of EM.

